# Highly Sensitive Temperature Sensor Based on Coupled-Beam AlN-on-Si MEMS Resonators Operating in Out-of-Plane Flexural Vibration Modes

**DOI:** 10.34133/2022/9865926

**Published:** 2022-08-20

**Authors:** Cheng Tu, Ming-hong Yang, Zi-qiang Zhang, Xiu-mei Lv, Lei Li, Xiao-Sheng Zhang

**Affiliations:** ^1^School of Electronic Science and Engineering, University of Electronic Science and Technology of China, Chengdu 611731, China; ^2^Beijing Xingfeng Aerospace Equipment Co., Ltd, Beijing 100854, China

## Abstract

This paper reports a type of highly sensitive temperature sensor utilizing AlN-on-Si resonators with coupled-beam structures of double- and triple-ended-tuning-fork (D/TETF). For both resonators, the out-of-plane flexural mode is adopted as it favors the effect of thermal mismatch between the composite layers inherent to the AlN-on-Si structure and thus helps attain a large temperature coefficient of resonant frequency (TCF). The analytical model to calculate TCF values of D/TETF AlN-on-Si resonators is provided, which agrees well with the finite-element simulation and experimental results. The resonant temperature sensor is built by closing the loop of the AlN-on-Si resonator, a transimpedance amplifier, a low-pass filter, and a phase shifter to form an oscillator, the output frequency of which shifts proportionally to the ambient temperature. The measured sensitivities of the temperature sensors using D/TETF resonators are better than -1000 ppm/°C in the temperature range of 25°C~60°C, showing great potential to fulfill the on-chip temperature compensation scheme for cofabricated sensors.

## 1. Introduction

With the rapid development of micro-electro-mechanical system (MEMS) technology, a variety of resonant MEMS sensors are emerging [1, 2]. Operation of MEMS devices hinges on energy conversion between different physical domains such as electrical and mechanical domains. According to the transduction mechanism, resonant MEMS sensors can be broadly divided into several types including capacitive [3], thermal/piezoresistive [4], and piezoelectric [5]. Piezoelectric resonant sensors have the advantages of large energy transduction efficiency, low power consumption, favorable frequency scaling characteristic, and quasidigital output [2]. However, conventional piezoelectric resonant sensors based solely on piezoelectric films have problems of low-quality factor (*Q*) and low power handling capability. To relieve these problems, Thin-film Piezoelectric-on-Substrate (TPoS) resonators composed of a piezoelectric film and underneath substrate layer have been proposed and gained widespread interest [6–11]. TPoS resonators combine the benefits of large transduction efficiency in piezoelectric thin films (such as AlN, PZT, and ZnO) and low acoustic loss in substrate materials (such as single crystal silicon, silica, and diamond), which ultimately results in low motional resistances and large *Q*s. Also, it has been reported that the power handling capability of TPoS resonators can be improved by increasing the thickness ratio of the substrate layer to the piezoelectric film [12]. All these advantages make the TPoS resonator a promising sensing unit in various sensor applications, such as mass sensor [6], inertial sensor [7], magnetometer [8], temperature sensor [9], humidity sensor [10], and liquid sensor [11].

Among various physical quantities to be measured, ambient temperature is the most important environmental factor that affects the performance of MEMS sensors. Since the structural dimensions of MEMS devices are usually in the range of micrometer to millimeter, their thermal time constants are small, which makes them vulnerable to temperature changes. One method to solve this issue refers to using microovens to keep the ambient temperature constant, which normally requires large power consumption [13, 14]. Another method is to accurately monitor the temperature fluctuations of the MEMS devices so that proper temperature compensation schemes can be fulfilled [15, 16]. This option usually requires a highly sensitive temperature sensor placed to the MEMS device as close as possible. An ideal solution refers to a temperature sensor using the same fabrication process as that of the MEMS device, which enables monolithic integration of both devices and thus minimizes the discrepancies caused by thermal gradients. So far, there has been a number of prior works showing the potential of using TPoS resonators for temperature sensing. Wu et al. reported several AlN-on-silica coupled-ring resonators operating in in-plane shear, radial breath, or extensional modes [17]. The reported devices exhibit temperature coefficient of frequency (TCF) ranging from 76 to 90 ppm/°C. The larger temperature sensitivity compared to the devices using silicon as substrate material (normally around -30 ppm/°C) is due to the higher temperature coefficient of Young's modulus of fused silica. Another interesting method to attain high |TCF| refers to the utilization of different temperature characteristics of two vibration modes. Fu et al. demonstrated such a AlN-on-Si rectangular resonator operating in both in-plane width-shear and width-extensional modes simultaneously [18]. Although the two modes exhibit TCF both around -30 ppm/°C, the small difference in their temperature characteristics has been utilized to realize high TCF of 1480 ppm/°C. The cost of this method is requirement of additional frequency processing circuits (e.g., multiplier and mixer), which serve to realize a linear combination of the resonant frequencies of two modes.

In this paper, two types of coupled-beam AlN-on-Si resonators using DETF and TETF structures both operating in the out-of-plane flexural vibration mode are analyzed for the application of temperature sensing. The reported devices utilize the out-of-plane flexural vibration mode to enhance the effect of thermal mismatch between the composite layers inherent to AlN-on-Si structures, which results in temperature sensitivities better than -1000 ppm/°C for both resonators using DETF and TETF structures.

## 2. Theoretical Model

### 2.1. Device Description

**Figure 1:**
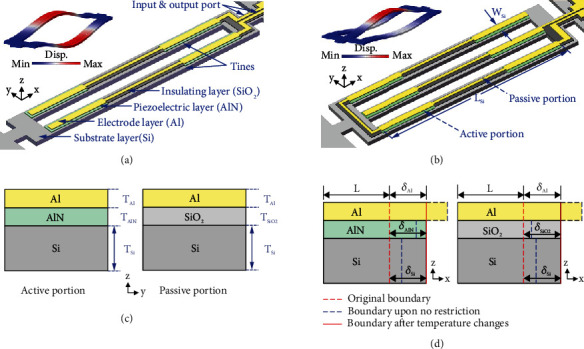
Perspective-view schematics and simulated out-of-plane flexural vibration mode shapes of the AlN-on-Si resonators based on (a) DETF and (b) TETF structures; (c) cross-sectional view of the structures in active and passive portions; (d) cross-sectional view of the structures showing 1D thermal deflections upon temperature increase.

Generally, the coupled-beam resonators have several beams (or tines) that are mechanically coupled via their common bases at two ends. The DETF resonator has two tines while TETF has three. Two typical coupled-beam AlN-on-Si resonators based on DETF and TETF structures are shown in Figures 1(a) and 1(b), respectively, together with their target first-order flexural out-of-plane vibration modes. To effectively excite the DETF and TETF resonators into their target modes, each tine is designed to have two portions, namely, active and passive portions, which are illustrated in Figure 1(c). The active portion comprises a piezoelectric layer (AlN) sandwiched by the electrode layer (Al) and substrate layer (Si). It should be noted that the top surface of the Si layer is highly doped and thus can serve as the “bottom electrode.” The active portion is responsible for piezoelectric actuation and sensing of the target mode. In the passive portion, the piezoelectric layer is replaced by a nonpiezoelectric layer (SiO_2_), which insulates the electrode and substrate layers. The active portion is placed close to both ends of a tine and constitutes 3/5 of the total length so that the piezoelectric transduction efficiency of the target mode is maximized [19]. In both DETF and TETF resonators under investigation, two-port configuration is adopted, which enables one tine for actuation and the other(s) for sensing.

The working principle of using an acoustic resonator as a temperature sensing unit relies on the shift in the resonant frequency of the resonator with temperature. In the AlN-on-Si resonators operating in flexural out-of-plane vibration modes, the frequency shifts with temperature are mainly caused by the difference in the thermal expansion coefficients of constituent layers. As the ambient temperature changes, thermal mismatch between constituent layers will result, causing an axial load on each tine, which in turn alters the axial stiffness of the resonator and thus shifts the resonant frequency. By monitoring the change of resonant frequency, one can obtain information on the ambient temperature.

### 2.2. Theoretical Analysis

In this section, a theoretical model is provided to characterize the temperature dependence of resonant frequency in AlN-on-Si resonators. This model is based on the method proposed in previous work [20]. Since the axial force plays the dominant role in determining the frequency shift in resonators operating in flexural modes, only the interaction force along the axis of the tines is considered in this model. As the temperature changes, the constituent layers will constrain each other to produce the same thermal deflection as shown in Figure 1(d). The thermal deflection (*δ*) of each layer can be expressed as

(1) $$\begin{array}{c} \delta_{\text{Si}}=L\alpha_{\text{Si}}∆T+\frac{F_{\text{Si}-\text{AlN}}\;L}{E_{\text{Si}}A_{\text{Si}}}, \end{array}$$

(2) $$\begin{array}{c} \delta_{\text{AlN}}=L\alpha_{\text{AlN}}∆T+\frac{\left(F_{\text{AlN}-\text{Al}}-F_{\text{Si}-\text{AlN}}\right)\;L}{E_{\text{AlN}}A_{\text{AlN}}}, \end{array}$$

(3) $$\begin{array}{c} \delta_{\text{Al}}=L\alpha_{\text{Al}}∆T-\frac{F_{\text{AlN}-\text{Al}}\;L}{E_{\text{Al}}A_{\text{Al}}}, \end{array}$$

where *L* denotes the original length, *A* is the cross-sectional area, *α* is the thermal expansion coefficient, *E* is Young's modulus, *F* represents the interaction force between adjacent two layers, and Δ*T* is the change of temperature. Since all three layers (Si, AlN, and Al layers) are held together physically, the actual deflections should be the same:

(4) $$\begin{array}{c} \delta_{\text{Si}}=\delta_{\text{AlN}}=\delta_{\text{Al}}. \end{array}$$

Substituting equations (1)–(3) into (4), one can obtain the force acted on the Si layer of active and passive portions (*F*_Si‐AlN_ and *F*_Si‐SiO_2__) as shown in the following equations:

(5) $$\begin{array}{c} F_{\text{Si}‐\text{AlN}}=k_{\text{Si}‐\text{AlN}}∆T, \end{array}$$

(6) $$\begin{array}{c} k_{\text{Si}‐\text{AlN}}=E_{\text{Si}}W_{\text{Si}}T_{\text{Si}}\frac{E_{\text{AlN}}\eta_{\text{AlN}}T_{\text{AlN}}\;\left(\alpha_{\text{AlN}}-\alpha_{\text{Si}}\right)+E_{\text{Al}}\eta_{\text{Al}}T_{\text{Al}}\left(\alpha_{\text{Al}}-\alpha_{\text{Si}}\right)}{E_{\text{Si}}T_{\text{Si}}+E_{\text{AlN}}\eta_{\text{AlN}}T_{\text{AlN}}+E_{\text{Al}}\eta_{\text{Al}}T_{\text{Al}}}, \end{array}$$

(7) $$\begin{array}{c} F_{\text{Si}‐\text{Si}{\text{O}}_{2}}=k_{\text{Si}‐{\text{SiO}}_{2}}∆T, \end{array}$$

(8) $$\begin{array}{c} k_{\text{Si}‐{\text{SiO}}_{2}}=E_{\text{Si}}W_{\text{Si}}T_{\text{Si}}\frac{E_{\text{Si}{\text{O}}_{2}}\eta_{\text{Si}{\text{O}}_{2}}T_{\text{Si}{\text{O}}_{2}}\;\left(\alpha_{\text{Si}{\text{O}}_{2}}-\alpha_{\text{Si}}\right)+E_{\text{Al}}\eta_{\text{Al}}T_{\text{Al}}\left(\alpha_{\text{Al}}-\alpha_{\text{Si}}\right)}{E_{\text{Si}}T_{\text{Si}}+E_{\text{Si}{\text{O}}_{2}}\eta_{\text{Si}{\text{O}}_{2}}T_{\text{Si}{\text{O}}_{2}}+E_{\text{Al}}\eta_{\text{Al}}T_{\text{Al}}}, \end{array}$$

where *k*_Si‐AlN_ and *k*_Si‐SiO_2__ denote the ratios of respective forces over temperature change, *W* and *T* are the width and thickness of the respective layer, and *η* denotes the coverage ratio of each layer with respect to the Si layer (e.g., *W*_Al_ = *η*_Al_ × *W*_Si_). Due to the fabrication limitation, *η*_Al_ < *η*_AlN_ < 1 (in active portion) and *η*_Al_ < *η*_SiO_2__ < 1 (in passive portion) should satisfy. Since the total thermal deflection of tine equals the sum of the thermal deflections of active and passive portions, it can be easily deduced that the equivalent axial force acted on the Si tine (*F*_Si_) is the weighted average of *F*_Si‐AlN_ and *F*_Si‐SiO_2__. Given that the active and passive portions take up 3/5 and 2/5 of the total length in the tine structure under investigation, the equivalent axial force can be expressed as

(9) $$\begin{array}{c} F_{\text{Si}}=\frac{-\left(3k_{\text{Si}‐\text{AlN}}+2k_{\text{Si}‐{\text{SiO}}_{2}}\right)∆T}{5}. \end{array}$$

Since the Si layer is normally much thicker than the other layers in AlN-on-Si resonators, it is assumed that the resonant frequency of the device can be calculated by only considering the axial force acted on the Si layer. Thus, the dependence of the resonant frequency of the first-order flexural mode on the axial force can be described by [21]

(10) $$\begin{array}{c} f_{s}=f_{n}\sqrt{1+F_{\text{Si}}\frac{0.295{L_{\text{Si}}}^{2}}{E_{\text{Si}}W_{\text{Si}}{T_{\text{Si}}}^{3}}}=f_{n}\sqrt{1+S_{\text{Si}}F_{\text{Si}}}, \end{array}$$

(11) $$\begin{array}{c} S_{\text{Si}}=\frac{0.295{L_{\text{Si}}}^{2}}{E_{\text{Si}}W_{\text{Si}}{T_{\text{Si}}}^{3}}, \end{array}$$

where *f*_*s*_ and *f*_*n*_ represent the resonant frequency with and without axial load, respectively, and *S*_Si_ denotes a factor determined by material properties and physical dimensions of the structure. Therefore, the first-order TCF can be obtained:

(12) $$\begin{array}{c} \text{TCF}=\frac{1}{f_{0}}\frac{df}{dT}=\frac{S_{\text{Si}}}{2\sqrt{1+S_{\text{Si}}F_{\text{Si}}}}\frac{F_{\text{Si}}}{∆T}=-\frac{S_{\text{Si}}}{10\sqrt{1+S_{\text{Si}}F_{\text{Si}}}}\;\left(3k_{\text{Si}‐\text{AlN}}+2k_{\text{Si}‐\text{Si}{\text{O}}_{2}}\right). \end{array}$$

A close observation of equation (12) suggests that TCF is dependent on Δ*T*, which means TCF is not strictly constant as temperature changes. However, the product of *S*_Si_ and *F*_Si_ is usually much smaller than 1, so TCF can be approximated as

(13) $$\begin{array}{c} \text{TCF}=-\frac{S_{\text{Si}}}{10}\;\left(3k_{\text{Si}‐\text{AlN}}+2k_{\text{Si}‐{\text{SiO}}_{2}}\right). \end{array}$$

Equation (13) indicates that the TCF can be approximated as a constant for a given structure since *S*_Si_, *k*_Si‐AlN_, and *k*_Si‐SiO_2__ are all determined just by material properties and physical dimensions as shown in equations (6), (8), and (11). Also, it can be seen from equation (13) that either increasing the length (*L*_Si_) or reducing the thickness (*T*_Si_) of the tine can improve TCF. However, mechanical reliability is of concern for the tine structure with a too large ratio of *L*_Si_/*T*_Si_. This work investigates two tine structures with different widths (*W*_Si_ = 20 *μ*m and 40 *μ*m) to verify the theoretical model.

It should be noted that the theoretical model described above is based on one single tine structure with two fixed ends. For coupled-beam structures such as DETF and TETF, mechanical coupling exists between the tines via their common bases, which will affect the axial loads on the tines. However, including the effect of the mechanical coupling between the tines will significantly complicate the model of computing TCF. Thus, this work only considers computing TCF of one single tine and assumes D/TETF structures have the same values of TCF.  Table 1 provides the calculated values of TCF for narrow and wide tines with *W*_Si_ = 20 *μ*m and 40 *μ*m, along with the physical dimensions and material properties used for each layer. It can be found that the wide tine has larger |TCF| compared to its narrower counterpart. This is mainly because the wider tine has a larger coverage ratio of Al layer (*η*_Al_) in the active portion, which results in larger *k*_Si‐AlN_ and thus larger |TCF| as shown in Table 1. The reason why the coverage ratio of the Al layer is important lies in the fact that Al has a much larger thermal expansion coefficient relative to other layers. It should be noted that the above theoretical analysis does not take into account the temperature coefficient of elasticity (TCE) of constituent materials. This is because the effect of thermal mismatch plays a dominant role in setting TCF for the devices under investigation than that from TCE. On this note, Si-based bulk mode MEMS resonators, which are dominated by the effect of TCE, usually exhibit TCF around -30 ppm/°C [9].

## 3. Results and Discussion

### 3.1. Simulation Results

**Figure 2:**
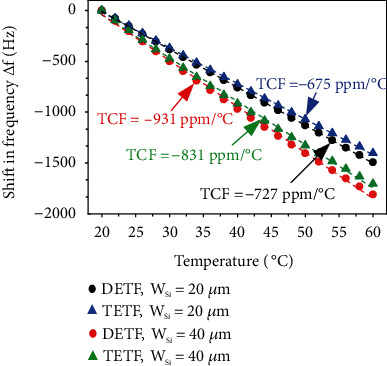
FE simulation results using thermal steady-state analysis showing dependence of resonant frequencies over temperature for four D/TETF resonators with *W*_Si_ = 20 *μ*m and 40 *μ*m.

To validate the theoretical model, a thermal steady-state analysis was performed based on coupled-domain finite element (FE) simulation using COMSOL Multiphysics. The coupled-domain FE simulation solves the associated coupled equations between the mechanical and thermal domains from which the shift in resonant frequency over temperature can be obtained. Note that the tine structures in the FE simulation model adopt the same physical dimensions and material properties as listed in Table 1.  Figure 2 shows the simulation results of four devices (D/TETF resonators with two different values of *W*_Si_). It can be seen from Figure 2 that the values of simulated values of TCF are close for D/TETF resonators with identical *W*_Si_, which suggests that the frequency temperature characteristics of D/TETF resonators are similar when the same tine structure is used. Also, it can be seen that D/TETF resonators with larger *W*_Si_ have larger values of |TCF|, which agrees well with the predictions from theoretical models. The good agreement between the simulated values of TCF of D/TETF resonators and the theoretical predictions based on one single tine structure suggests that TCF of D/TETF resonators can be estimated with reasonable accuracy by just considering the case of one tine. The discrepancy between the calculated and simulated results arises because the mechanical coupling between the tines is taken into account in FE simulation while not in the theoretical model.

### 3.2. Experimental Results

**Figure 3:**
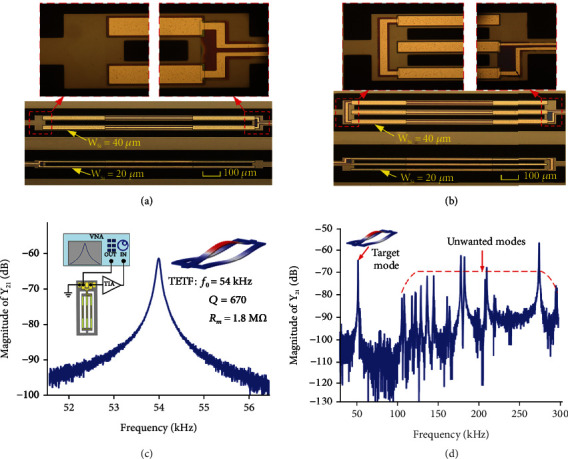
Optical micrographs of the fabricated AlN-on-Si resonators: (a) two DETF resonators with different values of tine width (*W*_Si_), (b) two TETF resonators with different values of *W*_Si_; measured two-port frequency response of transfer admittance *Y*_21_ for a TETF resonator with *W*_Si_ = 40 *μ*m in two different frequency spans: (c) small frequency span of 5 kHz showing the detail of target first-order flexural vibration mode with extracted *Q* and motional resistance (*R*_*m*_); (d) large frequency span of 270 kHz showing that the unwanted modes are at least 50 kHz away from the target mode.

Four D/TETF designs based on the narrow and wide tine structures (*W*_Si_ = 20 *μ*m and 40 *μ*m) were fabricated using a foundry AlN-on-SOI MEMS process. Figures 3(a) and 3(b) show the optical micrographs of four fabricated devices. The physical dimensions of the resonators are designed to be the same as that shown in Table 1. The measured two-port frequency response of transfer admittance *Y*_21_ for a TETF resonator is plotted in Figure 3(c), where the open-loop measurement setup is also provided. The resonant peak at 54 kHz corresponds to the target out-of-plane flexural mode. The extracted *Q* and motional resistance *R*_*m*_ of the resonator are 670 and 1.8 M*Ω*, respectively. Figure 3(d) plots the measured *Y*_21_ in a larger frequency span, showing the presence of other unwanted vibration modes above 100 kHz. To filter out the unwanted modes, a low-pass filter with sharp transition from passband to stopband is required.

All four D/TETF resonators under investigation were measured using the same experimental setup. It was found from the measured results that all four D/TETF resonators have similar resonant frequencies, which is expected since the resonant frequencies are mainly determined by the length and thickness of the tine structure. It was also found that, for devices with the same *W*_Si_, the peak values of TETF resonators were much larger than that of DETF ones. This is because the former has a larger transduction area, which results in lower motional resistance (*R*_*m*_). Since lower *R*_*m*_ of the resonator will alleviate the burden of amplifier design in an oscillator circuit, TETF resonators are preferred choices over DETF ones, especially considering that their values of TCF are similar as shown in the following section.

**Figure 4:**
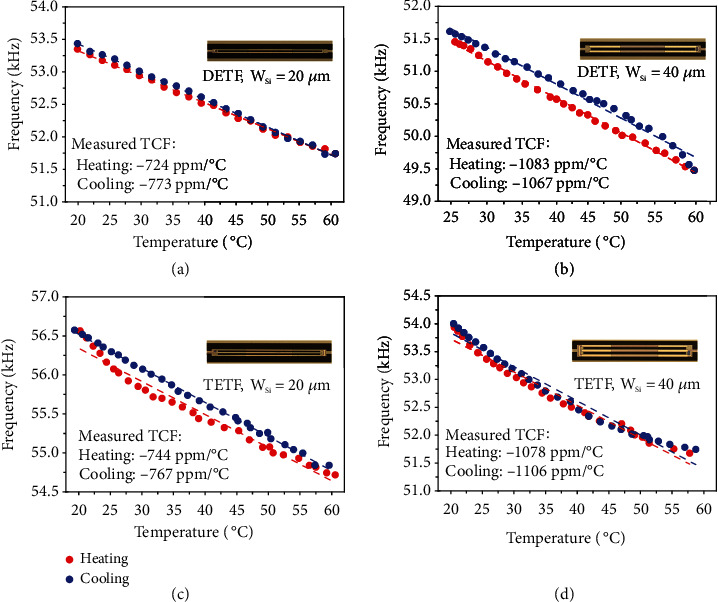
The measured temperature dependence of output frequencies in four oscillators during heating (in red) and cooling (in blue) for DETF resonators with (a) *W*_Si_ = 20 *μ*m and (b) *W*_Si_ = 40 *μ*m and TETF resonators with (c) *W*_Si_ = 20 *μ*m and (d) *W*_Si_ = 40 *μ*m.

The fabricated resonator was connected with the electronic circuits integrated into a PCB board to form an oscillator, the output frequency of which equals the resonant frequency of the resonator. By recording the shifts in the output frequency over temperature variations, one can measure TCF of the resonator which represents the temperature sensitivity of the resonant sensor. Figures 4(a)–4(d) show the measured output frequency over temperature for the oscillators using four fabricated D/TEFT resonators as the temperature sensing units. To confirm the repeatability of the measured results, a heating process was followed by a cooling process during each measurement. It can be seen that the output frequencies of oscillators shift almost in proportion to ambient temperature for all devices under test. Although small discrepancies exist for the measured data from the heating and cooling processes, they are much smaller than the total frequency shifts, which were used to extract the values of TCF. Please note that the observed small discrepancy probably arises from the inaccuracy in measured temperatures of the devices.

Table 2 compares the extracted values of TCF for the four D/TEFT resonators with that of calculated and simulated results, where good agreement can be seen. The measured results show that the D/TEFT resonators with wider tine structures (*W*_Si_ = 40 *μ*m) exhibit similar values of TCF, which are both better than -1000 ppm/°C. In comparison, the narrower D/TEFT resonators (*W*_Si_ = 20 *μ*m) show TCF values around 750 ppm/°C.

To test the long-term stability of the output frequency, Allan deviations of the oscillators were measured using the frequency counter at room temperature, the results of which are shown in Figure S1. It can be seen that the oscillators based on four D/TEFT resonators exhibit minimum frequency variations from 50 ppm to 150 ppm, corresponding to temperature resolutions from 0.05°C to 0.15°C.

## 4. Conclusion

In this work, we report a type of highly sensitive temperature sensor using an out-of-plane flexural mode D/TETF AlN-on-Si resonator as the temperature sensing unit. By utilizing the thermal mismatch between the composite layers inherent to the AlN-on-Si structure, the resonating sensors achieve large |TCF| which is better than 1000 ppm/°C.  Table 3 compares the performance of the state-of-the-art temperature sensors based on piezoelectric MEMS resonators. It can be seen that the temperature sensors reported in this work show large |TCF| compared to the previous work, eliminating the need for adding frequency processing circuits as required in [18, 22]. This advantage makes D/TETF AlN-on-Si resonators promising for realizing on-chip temperature sensors with smaller footprint and lower power consumption. Also, this work provides a theoretical model to calculate the TCF values of D/TETF AlN-on-Si resonators, which yields good agreement with FE simulation and measured results. Although demonstrated only for AlN-on-Si resonators, the theoretical model is considered applicable for all TPoS resonators using D/TETF structures, as long as the substrate layer is much thicker than the other layers and thus dominating the resonant frequency of the tine structure. Based on the theoretical model, this work shows that the TCF of D/TETF AlN-on-Si resonators can be estimated with reasonable accuracy by just considering one tine structure, which suggests that the mechanical coupling between the tines has little effect on frequency dependence on temperature. This interesting feature enables the enhancement of the transduction capability of the device by coupling more beam resonators without reducing TCF. The resonant temperature sensors reported in this work show great promise to realize the temperature compensation scheme for the cofabricated sensors also using TPoS structures.

## 5. Materials and Methods

### 5.1. Fabrication of AlN-on-Si Resonators

The resonators were fabricated using a foundry AlN-on-SOI process. The process starts with a silicon-on-insulator (SOI) wafer with a 10 *μ*m thick Si device layer, 1 *μ*m thick buried oxide layer, and 400 *μ*m thick wafer handle layer. First, the wafer is annealed in argon to drive the phosphorous dopant into the top surface of the Si device layer, which enables the realization of the top surface of the Si device layer as the “bottom electrode.” Then, a 200 nm thick oxide is thermally grown and patterned for electrical insulation. This is followed by reactively sputtering a 500 nm thick AlN layer, which is subsequently patterned by wet etching. A metal stack of Cr/Al (20 nm/1 *μ*m) is deposited and patterned to define the top electrodes, interconnection tracks, and contact pads. Next, deep reactive ion etching (DRIE) is used to etch the Si device layer to define the resonator structure. Finally, the handle layer is etched via DRIE from the backside of the SOI wafer, which is followed by wet etching of the buried oxide layer to release resonators. The fabrication process flow is provided in Figure S2.

### 5.2. Experimental Setup for Measuring *Y*_21_

The fabricated resonators were electrically characterized in a probe station using a vector network analyzer (VNA, Keysight N9914A). The measurement was performed in air at room temperature. Note that a transimpedance amplifier (TIA) with gain of 100 dB*Ω* is applied to the output of the resonator to improve the signal-to-noise ratio. This open-loop measurement setup is shown in Figure 3(c).

### 5.3. Experimental Setup for Measuring TCF

**Figure 5:**
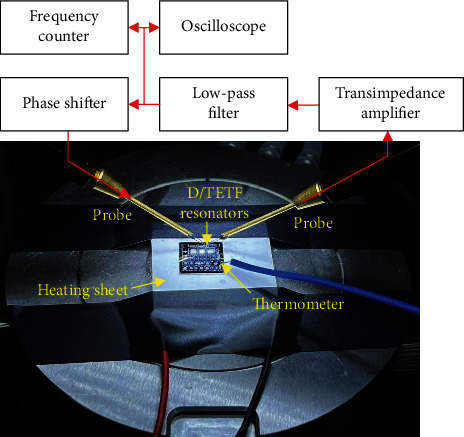
The schematic of the oscillator together with the measurement setup for characterizing the temperature dependence of resonant frequency in D/TETF resonators.

To implement an oscillator, the resonator was electrically connected with a TIA, followed by a low-pass filter and a phase shifter to form the close-loop, the schematic of which is shown in Figure 5. The output frequency of the oscillator is tracked to the resonant frequency of the D/TETF resonator. Note that the TIA, low-pass filter, and phase shifter are all integrated into a PCB board, which is electrically connected with D/TETF resonators via probes. The function of TIA is to convert the output current of the resonator to a voltage as well as compensate for the loss caused by the motional resistance of the resonators. To realize a low-pass filter with sharp roll-off, a fourth-order active filter based on the Sallen-Key topology is adopted. The phase shifter is implemented using an operational amplifier as an all-pass filter, which serves to provide the additional phase shift to fulfill the condition of Barkhausen criteria.

Figure 5 also shows the measurement setup for characterizing the temperature dependence of output frequency. A semiconductor heating sheet was used to heat the chip where the resonators were mounted. The ambient temperature of the chip was measured by a temperature recorder with model BCL-X. The thermometer of the temperature recorder was placed on the chip area close to the D/TEFT resonators. The output frequency of the oscillator was recorded by a frequency counter (RIGOL DG4202). It should be noted that stabilization of the output frequency was used as an indicator for device temperature approaching equilibrium with the ambient. This measurement setup allows characterizing the temperature dependence of output frequency of the oscillator in the range of 25°C to 60°C.

## Figures and Tables

**(a):** 

Symbol	Narrow tine	Wide tine
*L*_Si_ (*μ*m)	1300	1300
*W*_Si_ (*μ*m)	20	40
*η*_AlN_	0.7	0.8
Active portion *η*_Al_	0.4	0.65
*η*_SiO_2__	0.7	0.5
Passive portion *η*_Al_	0.4	0.25
*S*_Si_ (1/N)	146.63	73.32
*k*_Si‐AlN_ (N/°C)	1.23 × 10^−5^	3.79 × 10^−5^
*k*_Si‐SiO_2__ (N/°C)	1.08 × 10^−5^	1.36 × 10^−5^
Calculated TCF (ppm/°C)	-860	-1032
Simulated TCF (ppm/°C)	-727 (DETF)	-931 (DETF)
	-675 (TETF)	-831 (TETF)

**Table:** (b) Material properties and common physical dimensions

*α*_Si_ (1/K)	*α*_SiO_2__ (1/K)	*α*_AlN_ (1/K)	*α*_Al_ (1/K)
2.6 × 10^−6^	0.5 × 10^−6^	3.5 × 10^−6^	23.1 × 10^−6^
*E*_Si_ (GPa)	*E*_SiO_2__ (GPa)	*E*_AlN_ (GPa)	*E*_Al_ (GPa)
170	70	283	70
*T*_Si_ (*μ*m)	*T*_SiO_2__ (*μ*m)	*T*_AlN_ (*μ*m)	*T*_Al_ (*μ*m)
10	0.2	0.5	1

**Table 2:** Comparison of calculated, simulated, and measured values of TCF.

TCF (ppm/°C)	DETF *W*_Si_ = 20 *μ*m	DETF *W*_Si_ = 40 *μ*m	TETF *W*_Si_ = 20 *μ*m	TETF *W*_Si_ = 40 *μ*m
Calculated	-860	-1032	-860	-1032
Simulated	-727	-931	-675	-831
Measured (heating)	-724	-1083	-744	-1078
Measured (cooling)	-773	-1067	-767	-1106
Measured (mean)	-749	-1075	-755	-1092

**Table 3:** Performance comparison for temperature sensors using piezoelectric MEMS resonators.

Reference	[9]	[18]	[22]	This work	
Structure	AIN-on-Si plate resonator	AIN-on-Si plate resonator	AlN plate resonator	AIN-on-Si DETF resonator	AIN-on-Si TETF resonator
Vibration mode	S_0_ mode	WS & WE modes	S_0_ modes	Flexural mode	Flexural mode
Materials	Mo, AlN, Si	Mo, AlN, SiO_2_, Si	Mo, AlN	Al, AlN, SiO_2_, Si	Al, AlN, SiO_2_, Si
Dimensions *L* (*μ*m) × *W* (*μ*m)	≈100 × 100	204 × 156	≈350 × 350	1300 × 20/1300 × 40	1300 × 20/1300 × 40
Resonant frequency	990 MHz	27.56/27.58 MHz	180/500 MHz	54/52 kHz	57/54 kHz
TCF (ppm/°C)	-30	1480	334	-749/-1075	-755/-1092
Temperature range (°C)	20~85	-20~100	−25~100	25~60	25~60
Resolution (°C)	0.1	NA	NA	0.15/0.05	0.09/0.10

## Data Availability

The data used to support the findings of this study are available from the corresponding author upon reasonable request.

## Supplementary Materials

Supplementary Materials Figure S1: measured Allan deviations of four oscillators using D/TETF resonators with different *W*_Si_. Figure S2: fabrication process flow of the AlN-on-Si resonators.
