# Supplementary material for: Highly Sensitive Temperature Sensor Based on Coupled-Beam AlN-on-Si MEMS Resonators Operating in Out-of-Plane Flexural Vibration Modes
Source: Research (Wash D C). 2022 Aug 20;2022:9865926. doi: 10.34133/2022/9865926 (PMC9429980; doi:10.34133/2022/9865926)
Supplement: Supplementary Materials — Figure S1: measured Allan deviations of four oscillators using D/TETF resonators with different WSi. Figure S2: fabrication process flow of the AlN-on-Si resonators. [file 9865926.f1.docx]

**Supporting Information File**

Highly Sensitive Temperature Sensor based on Coupled-Beam AlN-on-Si MEMS Resonators Operating in Out-of-plane Flexural Vibration Modes

Cheng Tu^1^, Ming-hong Yang^1^, Zi-qiang Zhang^2^, Xiu-mei Lv^2^, Lei Li^1^ and Xiao-Sheng Zhang^1^*

1. School of Electronic Science and Engineering, University of Electronic Science and Technology of China, Chengdu 611731, China

2. Beijing Xingfeng Aerospace Equipment Co., Ltd, Beijing 100854, China

*Corresponding Author: [zhangxs@uestc.edu.cn](mailto:zhangxs@uestc.edu.cn) (XS Zhang)


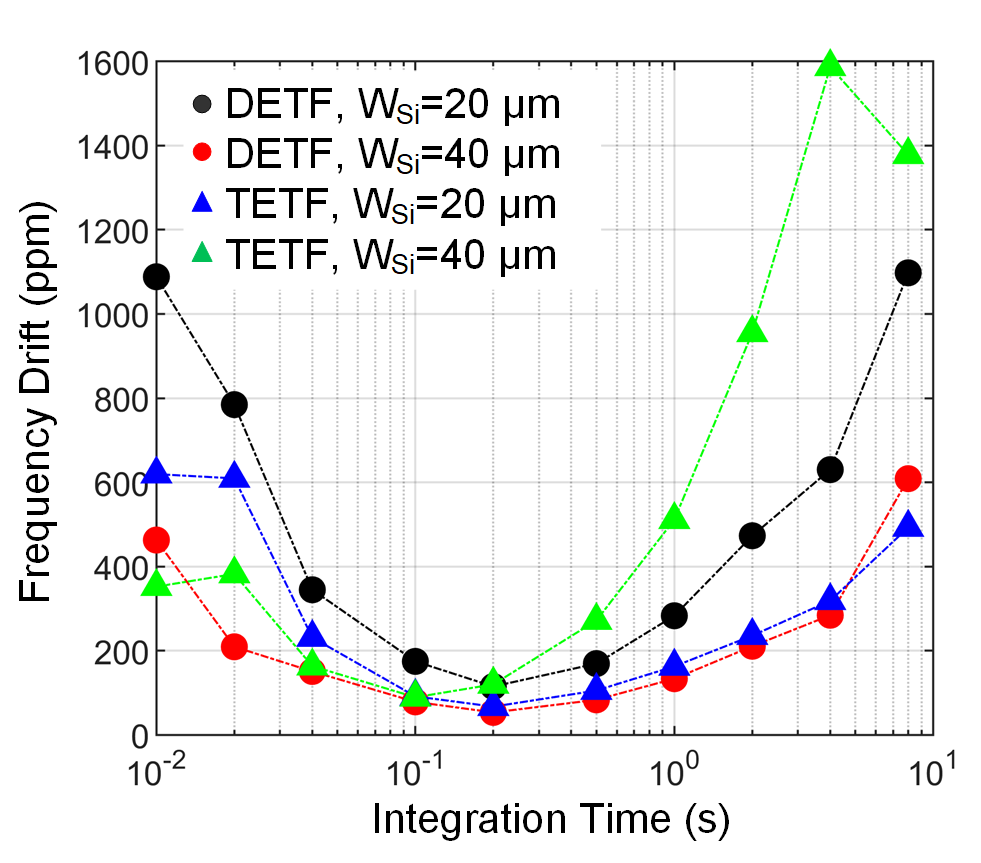


**Figure S1.** Measured Allan-deviations of four oscillators using D/TETF resonators with different *W_Si_*..


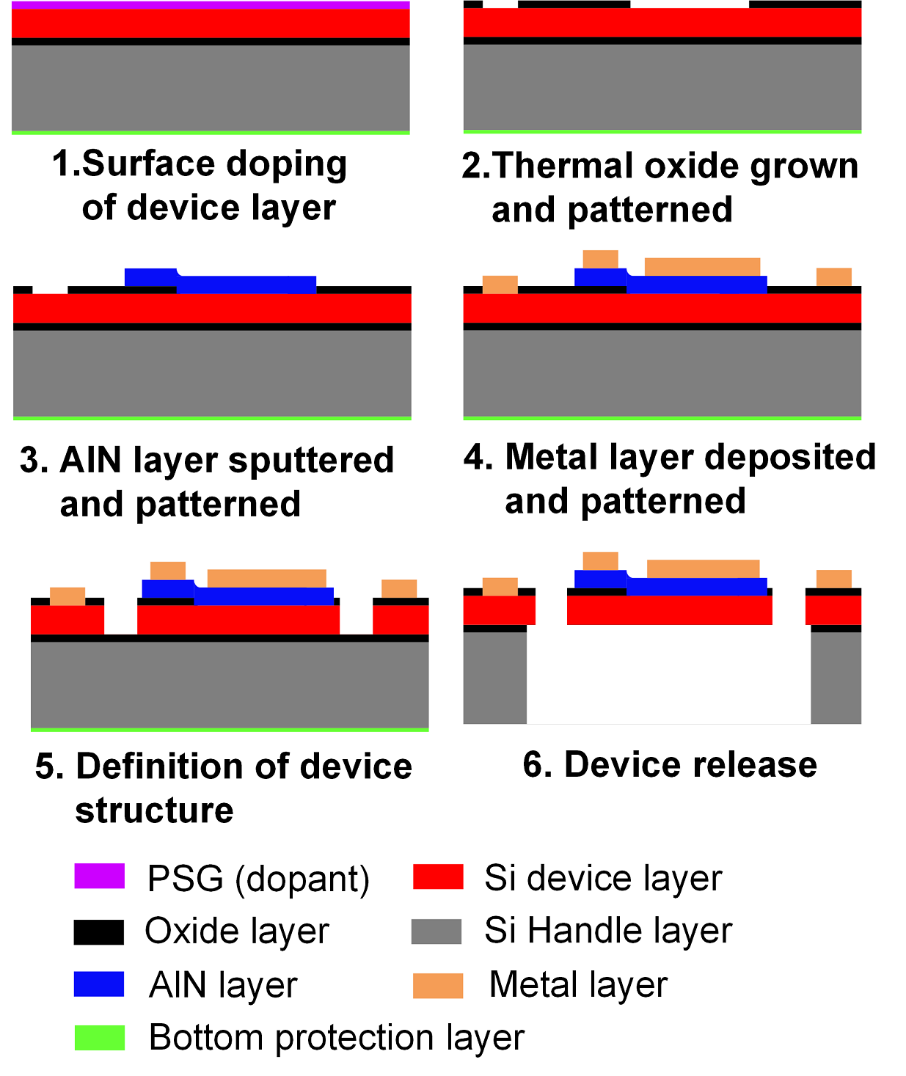


**Figure S2.** Fabrication process flow of the AlN-on-Si resonators.
